# Vaccine Advances against Venezuelan, Eastern, and Western Equine Encephalitis Viruses

**DOI:** 10.3390/vaccines8020273

**Published:** 2020-06-03

**Authors:** Zachary R. Stromberg, Will Fischer, Steven B. Bradfute, Jessica Z. Kubicek-Sutherland, Peter Hraber

**Affiliations:** 1 Physical Chemistry and Applied Spectroscopy, Chemistry Division, Los Alamos National Laboratory, Los Alamos, NM 505, USA; zrs@lanl.gov (Z.R.S.); jzk@lanl.gov (J.Z.K.-S.); 2 Theoretical Biology and Biophysics, Los Alamos National Laboratory, Los Alamos, NM 505, USA; wfischer@lanl.gov; 3 Center for Global Health, Division of Infectious Diseases, Department of Internal Medicine, University of New Mexico, Albuquerque, NM 505, USA; sbradfute@salud.unm.edu

**Keywords:** Alphavirus, antigens, DNA vaccine, Eastern equine encephalitis virus (EEEV), vaccine, Venezuelan equine encephalitis virus (VEEV), Western equine encephalitis virus (WEEV)

## Abstract

Vaccinations are a crucial intervention in combating infectious diseases. The three neurotropic Alphaviruses, Eastern (EEEV), Venezuelan (VEEV), and Western (WEEV) equine encephalitis viruses, are pathogens of interest for animal health, public health, and biological defense. In both equines and humans, these viruses can cause febrile illness that may progress to encephalitis. Currently, there are no licensed treatments or vaccines available for these viruses in humans. Experimental vaccines have shown variable efficacy and may cause severe adverse effects. Here, we outline recent strategies used to generate vaccines against EEEV, VEEV, and WEEV with an emphasis on virus-vectored and plasmid DNA delivery. Despite candidate vaccines protecting against one of the three viruses, few studies have demonstrated an effective trivalent vaccine. We evaluated the potential of published vaccines to generate cross-reactive protective responses by comparing DNA vaccine sequences to a set of EEEV, VEEV, and WEEV genomes and determining the vaccine coverages of potential epitopes. Finally, we discuss future directions in the development of vaccines to combat EEEV, VEEV, and WEEV.

## 1. Introduction

Vaccinations provide an effective means of protection from infectious diseases for humans and animals. Nevertheless, reactive vaccine developments are driven only by outbreaks, and epidemics leave populations at-risk for re-emerging diseases. This has been highlighted by recent epidemics by the arthropod-borne viruses (arboviruses) Chikungunya [1] and Zika [2]. The coronavirus vaccine development is another example, where an investment in a forward-looking vaccine design could help to prevent new outbreaks, rather than responding only when the latest viral outbreak emerges as a threat to global health, which delays the development of an effective vaccination countermeasure [3,4,5,6,7].

Arboviruses have emerged and re-emerged sporadically for centuries, but recent decades have seen increased rates of geographic dispersal due to factors such as growth of global transportation, urbanization, and failure of mosquito control [8]. In addition, by 2050, it is estimated that half of the world’s population will live in tropical environments, which altogether favors the re-emergence of these viruses [9].

Alphaviruses are members of the family *Togaviridae;* that contain enveloped positive-sense single-stranded RNA viruses, approximately 70 nm in diameter. Alphaviruses are often classified based on where they were originally isolated as either New- or Old-World viruses. New-World Alphaviruses, Eastern (EEEV), Venezuelan (VEEV), and Western (WEEV) equine encephalitis viruses circulate between rodents or birds and mosquito vectors and can spill over into equine and human populations [10]. In humans, these arboviruses cause disease of variable severity, ranging from mild febrile illness to life-threatening encephalitis [11]. Patients who survive infection may suffer long-term reduced quality of life, as well as high financial burdens; the lifetime cost to a person who suffers residual sequelae from EEEV infection can be several million US dollars [11,12].

Although in most cases EEEV results in a self-limiting illness, it can cause severe encephalitis with 30–75% mortality in humans, depending on age, and up to 90% in horses [11]. Symptoms include fever, vomiting, respiratory complications, and focal neurological symptoms. Death is rapid, within 3–5 days after infection, and 50–90% of survivors can have long-lasting neurological defects. EEEV was identified in 1933 as a separate virus from WEEV by serology [13]. At least 285 cases have been reported in the US alone since 1964 [11]; there is a cyclical pattern of reported cases in the USA, with a recent peak in 2019. Interestingly, there have been reports of EEEV and VEEV coinfections in humans and equines [14]. EEEV cases are most frequently reported in eastern North, South, and Central America.

VEEV was first discovered in equines (horses, donkeys, and mules) in 1936 after an investigation into sick horses in Venezuela [15]. Human VEEV infection was first reported in the 1960s, where a connection with mosquitos as the transmitting vector was described, as were cases of laboratory-acquired infections [16]. Since that time, there have been hundreds of thousands of human infections by VEEV in the Americas. Large individual outbreaks in humans with VEEV have been reported, such as in 1995 in Columbia, which resulted in ~75,000 infections, 3000 cases of neurological sequelae, and 300 deaths. A 1971 outbreak in Texas infected 86 individuals, and at least 12 had long-term neurological complications [17]. In the 1960s in Columbia [18], approximately 200,000 human cases of VEEV occurred, along with lethal infection in 100,000 equines [19]. VEEV infection in humans often results in a self-resolving mild flu-like syndrome. In a subset of patients, severe disease can occur, beginning with flu-like symptoms and progressing to encephalitis with high mortality. Overall, 1–4% of VEEV patients develop neurological symptoms, which can cause lethality or lifelong damage; additionally, birth defects and fetal demise can occur in infected pregnant women.

WEEV was discovered in 1930 during an encephalitis outbreak of thousands of horses and mules in California, where roughly half of the infected animals died [20]. WEEV initial infection in humans is usually mild but has a lethality rate of 3–7%, and up to 50% of survivors have permanent neurological symptoms. Infants and very young children have the highest risk of developing neurological sequelae after WEEV infection. These neurological complications include seizures, depression, paranoia, weakness, hearing loss, anxiety, speech disorders, and intellectual defects. WEEV infections occur primarily in the Central and Western United States. Over 1250 human WEEV cases have been reported.

Humans have generally been considered dead-end hosts, but recent evidence suggested that they may develop sufficiently high-titer viremia to continue the transmission cycle for VEEV [21]. Furthermore, concerns have been raised over the possibility that these pathogens, particularly EEEV and VEEV, could be transmitted as aerosols, making them potential bioterror agents [22]. Thus, the Centers for Disease Control and Prevention designated these pathogens as Category B organisms because of the ease of dissemination and the substantial morbidity associated with infection. There is an urgent need for a human vaccine that can be used for biothreat defense and public health, as well as protection for the agricultural community [23]. A trivalent vaccine that protect against EEEV, VEEV, and WEEV would greatly reduce the biological threat posed by these viruses.

EEEV, VEEV, and WEEV each induce outbreaks in distinct geographical regions. Previously, EEEV was classified as either North American or South American, but recently, South American EEEV has been reclassified as a Madariaga virus, based on studies suggesting that it is genetically distinct from North American EEEV [24]. This review will focus primarily on vaccines targeting North American EEEV. Cases of EEEV infection have been documented along the Gulf of Mexico from Texas to Florida and along the Atlantic Coast from Florida to Canada [25]. VEEV outbreaks have primarily been found in Central and South America but have spread as far north as the Southern US [10], whereas cases of WEEV are known from the Western US [25]. The geographical expansion of encephalitic Alphaviruses is particularly problematic in regions where host populations are entirely immunologically naïve [26,27].

Despite the fact that veterinary vaccines are widely available [28], there are currently no vaccines against EEEV, VEEV, or WEEV licensed for human use. Early research on vaccines against equine encephalitis viruses focused on the development of inactivated viruses through common methods such as heat or formalin [29]. Viruses were propagated in experimentally infected mice, horses, or chick embryos, isolated from infected tissues (brains), and subsequently inactivated [30]. In 1961, the US Army developed a live-attenuated vaccine called TC-83 and, in 1974, a formalin-inactivated vaccine called C-84 [31,32]. These vaccines have been used exclusively for laboratory and military personnel at risk for contracting VEEV and to immunize horses. TC-83 was developed by passaging of the virulent Trinidad donkey strain 83 times through guinea pig heart cell cultures [33]. This serial passage resulted in mutations in the 5′ noncoding region, nsp3, E2, E1, and 3′ noncoding region [34,35]. The current live-attenuated TC-83 can be transmitted by mosquitos and causes adverse side effects in ~20% of recipients [31]. TC-83 also has a high rate (~18%) of serological nonresponse [31] and (worryingly) has the potential to cause pancreatic disease [36] and teratogenic effects [37]. C-84, though associated with lower reactogenicity than TC-83, provides only short-term immunity in animal models and fails to protect against an aerosol challenge in hamsters [38]. It is therefore used only as a booster for TC-83 non-responders [39]. Furthermore, the incomplete inactivation of a VEEV equine vaccine caused an epidemic from 1969 to 1972, highlighting the potential dangers of live-virus vaccines [40]. The investigational EEEV vaccine TS-GSD 104 and WEEV vaccine TS-GSD 210 were developed from attenuated strains and formalin-inactivated [41]. Same-day administration of these two investigational vaccines resulted in immune interference in humans [41]. Immune interference was also found when TS-GSD 104, TS-GSD 210, and TC-83 were used sequentially to immunize human volunteers [42]. Thus, the impact of immune interference should be considered when developing multivalent vaccines. From the perspectives of biodefense and costs associated with severe illness, there is an urgent need for the development of an improved vaccine against EEEV, VEEV, and WEEV that is well-tolerated and highly immunogenic [23]. Here, we review recent progress made towards safe and immunogenic vaccines.

## 2. Animal Models and Strain Selection for Vaccine Evaluation

### 2.1. Animal Models

The usefulness of animal models to study any infectious disease relies on the ability of the model to reproduce characteristics of human disease. Readers are directed to an in-depth review describing animal models of Alphavirus encephalitis [43]. Briefly, horses are natural hosts and highly susceptible to epizootic strains, but they are unsuitable for routine vaccine testing due to their size, the veterinary expertise required for their use in experimental studies, and cost. Hamsters, rabbits, and guinea pigs have been used to a lesser extent. Of these animals, hamsters are the best-described and develop high titers of EEEV and WEEV in the brain, but after a subcutaneous infection with VEEV, some hamsters succumb to infection before central nervous system disease develops [44]. Mouse models have been studied extensively and can be used to study biodefense (aerosol infection) and mosquito transmission (subcutaneous infection). Although the subcutaneous route is traditionally administered via needle-inoculation, recent evidence from other viruses has suggested that the infection from a mosquito bite differs from a needle-inoculation in key features such as tissue tropism and replication kinetics [45]. Most nonhuman primate studies have used cynomolgus macaques, which are anatomically and immunologically similar to humans and susceptible to various routes of infection, including subcutaneous injection and exposure to aerosols.

### 2.2. Viral Strain Selection

Only a few challenge strains can feasibly be used when determining vaccine efficacy. Rationale for strain selection can depend on access to select-agent facilities (some strains are select agents; some are not) or access to BSL-3 laboratories (some BSL-2 vaccine strains of VEEV are available). Viral strains or isolates can be derived from human patient samples, equids, rodents, or mosquitos and may be selected based on the type of experiment being conducted. Rusnak et al. [46] described criteria for VEEV strain selection and propagation in detail based on the US Food and Drug Administration Animal Rule guidelines, Department of Defense vaccine requirements, strain availability, and experience generating challenge agents within the Filovirus Animal Nonclinical Group. Similar criteria were described by Wolfe et al. [23] for trivalent vaccine candidates: (1) strains from human clinical cases or those known to be capable of causing human disease, (2) strains with a low and well-characterized passage-history and that have been sequenced periodically to identify any pertinent changes to the genome or individual genes during cell passages in mammalian or insect cells [47], (3) relevant strains that are currently circulating, (4) strains that mimic a disease state in humans, (5) strains that are accessible to laboratories authorized to work with select agents, and (6) a panel of strains to test the breadth of protection [23,46]. Frequently used strains include VEEV Trinidad donkey, EEEV FL93-939, WEEV 71V-1658, and WEEV CBA-87.

## 3. Vaccine Strategies

Currently, there are several vaccines under development to protect against encephalitic Alphaviruses. Candidate vaccines have been developed using various approaches: live-attenuated virus, inactivated virus, passive immunization, replicon particles, viral vectors, and DNA; vaccine strategies against VEEV were recently reviewed by Sharma and Knollmann-Ritschel [48]. While multiple types of vaccine platforms are used to combat infection of a wide range of pathogens, there are considerations to be taken into account when designing vaccines for pathogens of special interest. Live-attenuated viruses are often immunogenic, induce both cell-mediated and humoral responses, and do not require adjuvants to be effective. However, these vaccines may induce side effects, and some studies have shown that mosquitos can acquire attenuated Alphaviruses from vaccinated equids [49,50].

The safety and stability of live-attenuated vaccines has been dramatically improved through rational design using techniques including codon-deoptimization, gene rearrangement, and targeted attenuating mutations. Codon-deoptimization, the replacement of commonly used codons with rare codons, can significantly reduce protein translation, which has been shown to enhance the attenuation of several live viral vaccines [51,52,53,54]. Similarly, gene order in a viral genome is highly conserved, and the rearrangement of some key genes can greatly reduce viral growth rates [55]. Attenuating mutations have been the most common method for reducing the virulence of live vaccine strains, but some mutations are more stable than others. The rational selection of highly stable attenuating mutations has significantly increased viral vaccine safety [56]. DNA-launched live-attenuated vaccines are a promising technology showing an improved safety, stability, and efficacy of RNA viruses by encoding the complete genome in a DNA plasmid that is then transcribed by the host cells in vivo [57]. This technology has been used to improve the safety of the VEEV TC-83 vaccine by introducing the stable attenuating mutation (E2-T120A) in the viral glycoprotein [58], as well as the rearrangement of structural genes such that the capsid is expressed downstream of the glycoprotein [59].

**Figure 1:**
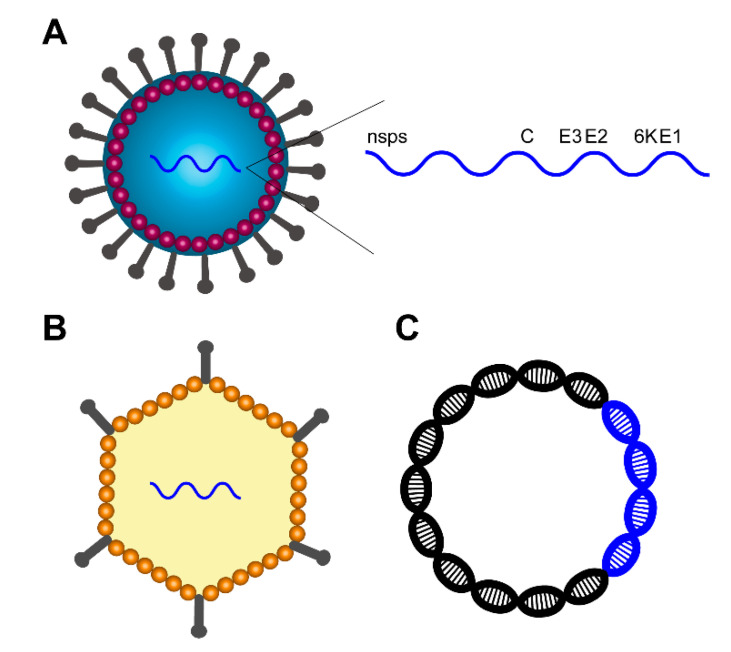
Vaccine strategies against encephalitic Alphavirus infections. (**A**) Schematic overview of encephalitic the Alphavirus structure, including the genome organization of nonstructural proteins (nsps), capsid (**C**), assembly protein (E3), spike glycoproteins (E2 and E1), and viroporin protein (6K). (**B**) Viral vector delivery of encephalitic Alphavirus genes (blue). (**C**) Plasmid DNA containing backbone (black) and encephalitic Alphavirus DNA (blue).

Inactivated vaccines are generally safe (unless the inactivation is incomplete, as has likely occurred with VEEV [40]) but may induce short-lived immunity that requires frequent booster shots. Passive immunization with antibodies (often derived from prior immunization) can protect against infectious virus challenge, but antibody targets should be considered, as protection can differ depending on the targeted epitope [60,61]. Another strategy uses alphavirus replicon particles derived by deleting genes encoding structural proteins such as the capsid; these have shown to be effective both as individual and trivalent vaccines against EEEV, VEEV, and WEEV [62]. Viral-vectored vaccines have the advantage of inducing robust cell-mediated responses in addition to antibody generation due to their ability to generate target antigens inside infected cells. However, these vaccines may have a higher risk of inducing side effects [4], and in some cases, there may be a high level of pre-existing immunity to the vector itself in target populations [63]. Plasmid DNA vaccines are generally safe and can induce both antibody and T cell responses but tend to induce short-lived responses and may require multiple boosts. The flexibility to rapidly generate novel sequences matching viral outbreak strains is a major advantage of plasmid DNA vaccines. This review focuses on viral vector and plasmid DNA vaccines as newer approaches for combating encephalitic Alphaviruses (Figure 1).

## 4. Recent Progress in the Development of Viral Vector Based Vaccines

Viral-vectored vaccines can express heterologous (different strain of the same virus) antigens and induce strong antigen-specific immune responses. A Vaccinia virus expressing a hepatitis B antigen was the first viral vector developed [64]; subsequently, numerous viruses including adenovirus, canarypox, herpesvirus, lentivirus, and others have been explored for use as viral vectors [65]. In some cases, viral-vectored vaccines can provide long-term immunity after a single dose [66]. Many viral vectors are considered live vaccines, but replication is often deficient or attenuated to increase safety; however, a reversion to virulence is often a concern. Thus, the use of safe viral vectors to deliver structural genes has gained interest. In this section, we discuss various viral vectors for the delivery of antigens of encephalitic Alphaviruses. Table 1 provides a summary of recent vectored vaccines.

### 4.1. Adenovirus Vector

Adenovirus vectors are highly immunogenic, effective carriers of foreign antigens [82] and have been explored for protection against VEEV and WEEV in mouse models. The human type 5 adenovirus was used to produce E3-E2-6K of the VEEV strain TC-83 in the RAd/VEEV#3 vaccine [67]. For vaccines delivering TC-83 genes, a challenge with Trinidad donkey was considered a homologous strain. An immune response to a homologous challenge, which matches the vaccine strain, is less of an accomplishment than to heterologous challenge, which may indicate cross-protective responses. RAd/VEEV#3 provided almost complete protection (90%) against a homologous challenge with Trinidad donkey, but when immunized mice were challenged with heterologous strains, a variable protection (50–100%) was observed. RAd/VEEV#3 was further explored with the coadministration of CpG [68], but CpG adjuvantation did not provide significantly better protection than RAd/VEEV#3 alone and resulted in antibody responses directed against the adenovirus vector. The E3-E2-6K sequence was codon optimized for expression in a mammalian host in the RAd/VEEV#3-CO vaccine, which provided significantly better protection against homologous challenge than the nonoptimized vaccine [69].

The human adenovirus type 5 was also used to deliver WEEV antigens. Two studies evaluated an adenovirus vector producing E3-E2-6K-E1 (Ad5-WEEV) from the WEEV strain 71V-1658 [70,71]. Initially, two doses of Ad5-WEEV were used to provide complete protection against a homologous challenge [70]. In a follow-up study, a single-dose of Ad5-WEEV provided complete protection against a homologous challenge and partial-to-complete protection against a heterologous WEEV challenge. To determine if the adenovirus vector delivery of E1 alone could provide protection against WEEV, Ad5-E1 was constructed using the sequence of 6K-E1 of 71V-1658 [72]. A single-dose of Ad5-E1 provided complete protection against a homologous challenge, while a heterologous challenge resulted in no-to-complete protection depending on the number of days that the challenge occurred postvaccination. Overall, the robustness of adenovirus-vectored vaccines needs improvement to protect against heterologous strains.

### 4.2. Eilat Virus Vector

Alphavirus vectors can be used to deliver antigens derived from bacteria, parasites, and viruses, including Alphaviruses of the same or different species [83]. Recently, the Eilat virus (EILV), an Alphavirus with a host range restricted to insects [84], was used to deliver the structural proteins C-E3-E2-6K-E1 of EEEV and VEEV [73]. A single dose of EILV/EEEV or EILV/VEEV completely protected against homologous and heterologous challenges, respectively. In the same study, a blending of equal parts EILV/EEEV, EILV/VEEV, and EILV expressing the structural proteins C-E2-E1 from the Chikungunya virus provided 80% protection against EEEV and 90% protection against VEEV challenges. It was suggested that reduced efficacy with the multivalent vaccine compared to monovalent vaccines may reflect an immune interference [73]. EILV has favorable characteristics as a vector, such as the inability to replicate in vertebrates, which makes it an intrinsically safe approach. Although several insect-specific viruses have been identified [85,86], they have largely been ignored for use as vaccine vectors, but their applications in limited studies show great promise [73,87].

### 4.3. Equine Herpesvirus Vector

Equine herpesvirus type 1 (EHV-1) presents suitable characteristics to be a universal vaccine vector: it can enter a wide variety of cell types, can accept large amounts of foreign DNA, and is easily maintained and manipulated [88]. The vaccine candidate rH_VEEV used EHV-1 strain RacH to deliver a codon-optimized sequence of E3-E2-6K-E1 from TC-83 [74]. Immunization of mice with rH_VEEV conferred no protection with the lowest vaccine dose (10^2^ plaque-forming units [PFU]) up to complete protection with the highest vaccine dose (10^4^ PFU) after challenge with a heterologous VEEV strain. Neutralizing antibodies were not detected, leading the authors to speculate that T cell responses or antibody-mediated protection unrelated to neutralizing activity played a role in protection. A better mechanistic understanding of rH_VEEV would be valuable for developing improved vaccines based on rH_VEEV.

### 4.4. Isfahan Virus Vector

Isfahan virus (ISFV) of the genus *Vesiculovirus* resides in sandflies and can be transmitted to humans but has not been conclusively associated with illness [89]. Vaccine candidates rISFV-EEEV and rISFV-VEEV were developed by delivering foreign antigens E3-E2-6K-E1 in an ISFV strain [75]. The vaccines were used alone or as a bivalent mixture conferring 100% protection to homologous challenge in a mouse model; protection was likely mediated by neutralizing antibodies. Future studies should investigate the usefulness of this vaccine against heterologous challenges.

### 4.5. Sindbis Virus Vector

The Sindbis virus (SIN) is an Alphavirus belonging to the WEEV complex that was first isolated from mosquitoes in Egypt and is one of the least pathogenic Alphaviruses known [90]. Chimeric SIN/EEEV, SIN/VEEV, and SIN/WEEV viruses have been investigated for use as live virus vaccines. In one study, Sindbis virus was used for the nonstructural genes, and the EEEV structural genes C-E3-E2-6K-E1 were derived from North American (NA) strain FL93-939 to create the vaccine SIN/NAEEEV [76]. After immunization with SIN/NAEEEV, mice developed high titers of neutralizing antibodies. Following challenge with FL93-939, 80–100% of mice vaccinated with SIN/NAEEV survived, depending on the vaccine dose. This vaccine was also evaluated in a cynomolgus macaque model with a lethal EEEV aerosol challenge [77]. Most (82%) SIN/NAEEEV vaccinated macaques survived with little evidence of disease.

Four candidate Sindbis viral vaccines: SIN-83, SAAR/TRD, SIN/TRD, and SIN/ZPC have been developed against VEEV. SIN-83 was constructed using structural genes C-E3-E2-6K-E1 from the VEEV vaccine strain TC-83 [78]. Mice immunized with SIN-83 developed neutralizing antibodies at slightly lower titers than TC-83 vaccination but did not cause detectable disease in mice. The SIN-83 vaccination completely protected against the intranasal and subcutaneous and partially protected against the intracerebral challenge with heterologous VEEV strain ZPC 738 [78,79]. Additional chimeric vaccines SAAR/TRD and SIN/TRD expressing structural proteins from Trinidad donkey and SIN/ZPC-expressing structural proteins from ZPC 738 were created [79]. All three vaccines protected 100% of mice and hamsters challenged with VEEV strain ZPC 738. Safety is a concern with any live vaccine, and these vaccines are no exception. In a six-day-old mouse model of VEEV central nervous system infection, vaccination with the standard vaccine, TC-83, was lethal—100% of the vaccinated mouse pups died; however, intermediate survival (60–80%) was observed for vaccination with SAAR/TRD, SIN/TRD, or SIN/ZPC, and all mice survived vaccination with SIN-83 [79].

The Sindbis virus has also been used as a vector for the delivery of WEEV structural proteins. Three vaccine candidates were constructed against WEEV, including SIN/CO92, SIN/SIN/McM, and SIN/EEE/McM [80]. After a challenge with WEEV strain McMillan, 40–100% of mice vaccinated with SIN/CO92 survived, depending on the vaccine dose. In contrast, all mice vaccinated with SIN/SIN/McM or SIN/EEE/McM survived. This provides another example of improved postchallenge survival when mice are immunized with structural proteins derived from the same strain that is used for challenge.

### 4.6. Vaccinia Virus Vector

Modified vaccinia Ankara-Bavarian Nordic (MVA-BN) is a nonreplicating vector that has been extensively used to deliver antigens and is approved for use in a smallpox vaccine licensed in Canada and Europe [91], demonstrating the utility of this vector. In one study, MVA-BN was used to deliver E3-E2-6K-E1 of EEEV, VEEV, or WEEV in a monovalent vaccine, as a mixture of all three monovalent vaccines, or in a trivalent formulation [81]. The mixture of the three vaccines and the trivalent formulation are among the few viral vector vaccines that can provide simultaneous protection against EEEV, VEEV, and WEEV. The mixture and trivalent formulations provided significantly higher levels of protection compared to the empty vector as a control. Although immunization with the mixture resulted in a numerically higher percentage of survival for EEEV and WEEV challenges and protected equally well against VEEV challenge compared to the trivalent formulation, either the authors did not statistically compare the vaccinations against each other or they were not statistically significant. These data warrant further analyses to determine whether a single trivalent vaccine or a mixture of monovalent vaccines provides superior cross-protection against encephalitic Alphaviruses.

### 4.7. Vesicular Stomatitis Virus Vector

The Vesicular stomatitis virus can induce strong cellular and humoral immune responses and has been used as a vector to protect against several viral infections [92,93,94]. The same study that tested ISFV as a vector used vesicular stomatitis virus serotype Indiana (VSIV) as a vector to deliver E3-E2-6K-E1 from VEEV strain ZPC 738 [75]. Like the rISFV-VEEV vaccine, rVSIV-VEEV conferred 100% protection against a homologous challenge in a mouse model. Evaluating the ultimate utility of this vaccine will require further testing with heterologous challenge strains: a common theme for most vaccines.

## 5. Recent Progress in the Development of Plasmid DNA Vaccines

DNA vaccinations routinely deliver a DNA plasmid encoding one or more antigens to induce an immune response. DNA vaccinations have many advantages: ability to break the cold chain, easy manipulation, large-scale manufacturing, low cost, molecular stability, and no live components [95]. Breaking the cold chain is particularly important for field deployment and delivery in remote tropical areas. DNA vaccines may include adjuvants to stimulate the immune system and molecules to assist in cell targeting and entry. The vector design has been improved by reducing the size of bacterial backbone regions, such as in the use of bacterial RNA-based (antibiotic free) markers [96,97,98]. Strategies to optimize the expression of antigens include the addition of an optimal promoter, immunostimulatory sequences, localization and secretory signals, Kozak sequence, and codon optimization [99,100]. Drawbacks of DNA vaccines include the potential for autoimmune responses by eliciting anti-DNA antibodies, the unlikely integration into the host’s genome, extraneous mutations, and low immunogenicity, often requiring delivery by electroporation, the use of immunostimulatory adjuvants, multiple doses, and large amounts of DNA [101,102]. Genes encoding EEEV, VEEV, and WEEV antigens have been cloned into plasmid DNA vaccines and tuned for administration in mammalian hosts. Table 2 summarizes the viral strains and structural protein regions used for recent plasmid DNA vaccine candidates.

### 5.1. DNA Vaccines for EEEV

One recent study described the immunogenicity of a DNA vaccine targeting EEEV [103]. The pcDNA3.1-C-E vaccine included the structural genes C-E3-E2-6K-E1 of EEEV with expression facilitated by a cytomegalovirus (CMV) promoter. The pCDNA3.1-C-E vaccine induced antiviral cytokines, high levels of neutralizing antibodies, and T cell responses, but the EEEV challenge was not assessed. Future studies should assess the ability of pcDNA3.1-C-E to protect against EEEV challenges in an animal model.

### 5.2. DNA Vaccines for VEEV

The use of DNA vaccines has been more extensively studied to prevent VEEV infection, while only a few studies exist for DNA vaccines targeting EEEV and WEEV. Although several VEEV vaccine studies are listed in Table 2, the structural genes have been derived from only two strains, viz., Trinidad donkey and TC-83. The rationale for selecting these strains is likely in part because of convenience, the body of literature on TC-83 as a live attenuated vaccine, and viral vectors delivering structural proteins from these strains. Vaccines 26S [105] and VEEV DNA [108] used sequences corresponding to structural proteins from Trinidad donkey to create vaccine plasmids delivered via gene gun. The delivery of vaccine plasmids by electroporation (EP) or gene gun elicits a strong immune response and requires less DNA, but the tradeoff is often cell death due to its inherent invasiveness [95]. The 26S vaccine induced IgG antibodies and protected 100% of subcutaneous- and 80% of aerosol-challenged mice [105]. Furthermore, the VEEV DNA vaccine induced a humoral immune response and protected two of three macaques from serum viremia, whereas all control animals developed serum viremia in a nonlethal aerosol challenge [108]. Immunoinformatics was used to present tailored epitopes of VEEV antigens derived from Trinidad donkey in a multiepitope vaccine, which induced cellular and humoral immune responses but had a low level of protection against homologous challenge [113]. Directed molecular evolution was another approach used with the intent to improve the ability of the cross-protection of DNA vaccines derived from Trinidad donkey [107]. These vaccines (AG2-5A7, AG2-5A10, AG4-1C7, and AG-1G2) yielded 70–100% protection against a homologous challenge, but a heterologous challenge was not tested. Trinidad donkey sequences were codon-optimized to improve the protective efficacy of a VEEV DNA vaccine. The VEEV_COCAP_ and VEEV_CO_ plasmids were constructed using codon-optimized sequences of Trinidad donkey structural proteins with or without the capsid, respectively [109]. The capsid was removed from this vaccine and others, because it can be cytotoxic and inhibit translation [117]. Mice immunized with VEEV_CO_ had improved neutralization titers compared to VEEV_COCAP_, and it was subsequently used in other studies to evaluate the efficacy in combination with adjuvants [110] and in a Phase 1 clinical trial [111].

For plasmid DNA vaccines, sequences from TC-83 have also been explored. In one study, mice were immunized with a plasmid DNA vaccine composed in part of E3-E2-6K of TC-83 and boosted with the earlier described adenovirus vector RAd/VEEV#3 vaccine to improve immunogenicity [106]. This study found 83% protection against a homologous challenge but failed to assess a heterologous challenge and used four doses, which is suboptimal for a rapid response. A lack of a heterologous challenge is a major limitation in most plasmid DNA VEEV vaccine studies. In contrast, a DNA-launched live-attenuated vaccine of VEEV was created by creating a DNA plasmid encoding the complete genomic RNA of TC-83 CMV promoter and induced 100% protection against a heterologous challenge after a single dose [57,112]. The use of the whole genome in a DNA-based vaccine is a novel approach that generates a live virus and combines the properties of DNA and live attenuated vaccines.

### 5.3. DNA Vaccines for WEEV

Two studies have provided evidence that a DNA vaccine may be a promising approach against WEEV infections. The first study developed a plasmid, pVHX-6, containing the C-E3-E2-6K-E1 of WEEV strain 71V-1658 [114]. Four doses of pVHX-6 delivered intra-muscular with electroporation (IM-EP) and resulted in elevated cytotoxic T cell activity and complete protection against a homologous challenge. However, the level of protection dropped to 50–62% when immunized mice were challenged with heterologous WEEV strains (CBA87 or Fleming). In another study, plasmids were constructed from different portions of the structural genes from pVHX-6 (i.e., pE3-E2-6K-E1, pE3-E2, or p6K-E1) and analyzed for protection against a WEEV challenge [115]. In the absence of E1, the vaccine pE3-E2 failed to protect against a homologous challenge, but plasmids p6K-E1 and pE3-E2-6K-E1, which included E1, yielded 100% survival. Mice immunized with pE3-E2-6K-E1 were mostly protected against a heterologous challenge, but other vaccines provided limited protection. 

### 5.4. Trivalent DNA Vaccines

One trivalent DNA vaccine, 3-EEV, comprised E3-E2-6K-E1 from EEEV strain FL91-4679, VEEV strain Trinidad donkey, and WEEV strain CBA87 [116]. The 3-EEV vaccine elicited antibody responses against all three targets and induced complete protection against homologous EEEV, VEEV, and WEEV challenges. A challenge with a heterologous strain was not tested, but the potential for heterologous protection was assessed by measuring neutralization activity against a panel of heterologous strains. Altogether, only three of 13 DNA vaccine studies assessed protection against a heterologous challenge; easily assessing the breadth of DNA vaccines is an obstacle in this field.

## 6. Recent Progress in the Development of RNA Vaccines

RNA vaccines have several key advantages including heightened immunogenicity, lower dosing requirements, and improved antigen expression as compared to DNA vaccines, as well as enhanced safety and reduced manufacturing time over live-attenuated vaccines [118,119]. Sequence optimization and the use of modified nucleosides can increase the efficacy of RNA vaccines [120]. RNA can be delivered as a naked injection or encapsulated to reduced degradation. Recent advances using 1-methylpseudouridine nucleoside-modified RNA against other viruses exhibit greater stability, persistence, and immunogenicity profiles due to reduced degradation in vivo [121,122,123]. Drawbacks of RNA vaccines include unintended immune reactions and instability in vivo and during storage, as well as multiple dosing requirements to achieve sustained protection [119]. Self-amplifying mRNA technology utilizes synthetic RNA derived from the viral genome, which is then encapsulated in lipid nanoparticles or cationic nanoemulsions [124]. This technology has produced an mRNA vaccine encoding the genome of VEEV strain TC-83 that shows enhanced safety with similar immunogenicity as an inoculation with the live TC-83 strain and 100% protection against an aerosol VEEV challenge [125].

## 7. Evaluation of Candidate Vaccine Sequences

**Figure 2:**
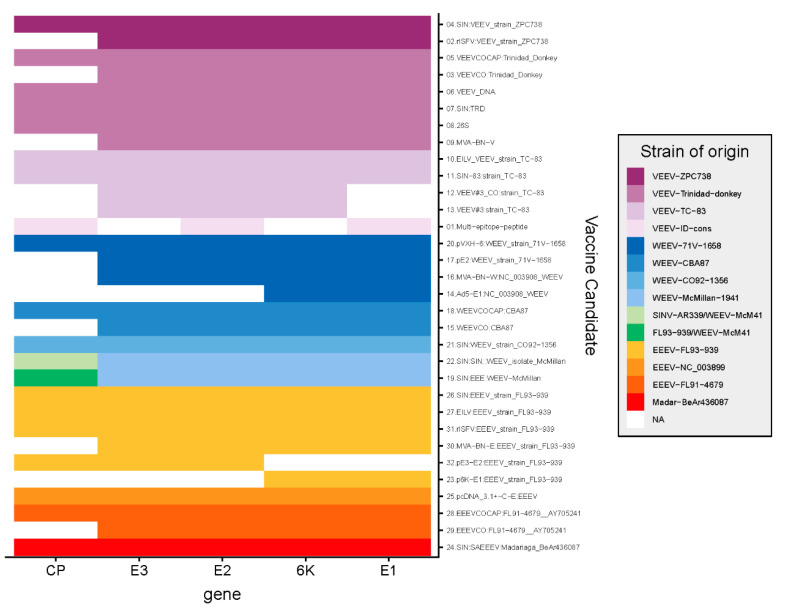
Strain origin and gene content of Alphavirus structural, protein-based, viral-vectored, and plasmid DNA vaccines. Venezuelan equine encephalitis virus (VEEV) vaccines are indicated in purple, Western (WEEV) vaccines in blue to green, and Eastern (EEEV) vaccines in orange to red; genes not included in a particular vaccine (NA) are indicated in white.

Vaccine antigens should elicit robust cross-reactive responses against circulating viruses. Generally, antigen selection is based on well-known virulence factors or computational discovery and assessed by in vitro and in vivo methods [126]. The common approach for the development of encephalitic Alphavirus vaccines is to deliver structural genes (Figure 2) from a reference strain or a strain in the laboratory collection, such as Trinidad donkey. Although this approach often provides protection against a homologous challenge, heterologous protection may be weak, because viral diversity was not considered.

**Figure 3:**
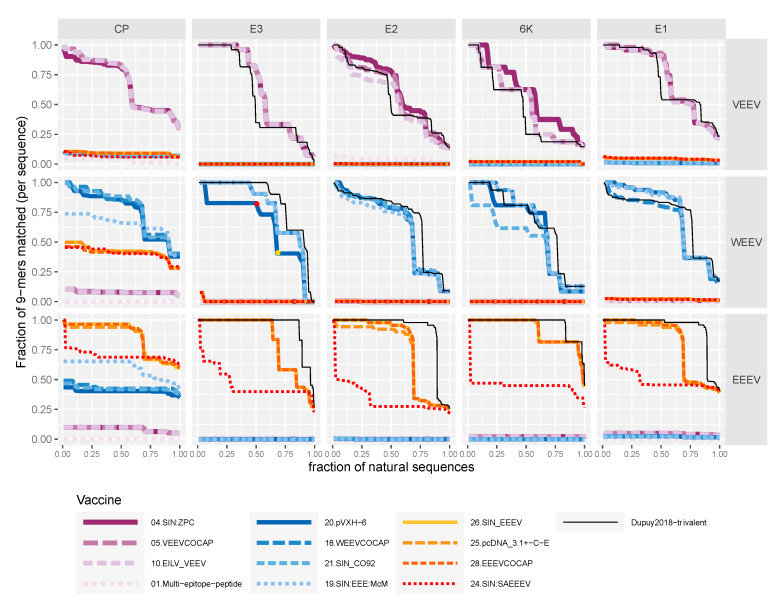
Matching of potential linear epitopes (9-amino-acid fragments) in natural sequences by selected vaccine candidates. Highly similar sequences were removed from an alignment of all available VEEV-clade, WEEV-clade, and EEEV-clade sequences in GenBank in November 2019. For each vaccine, the fraction of vaccine-matched 9-mers, “9-mer coverage”, was computed for each sequence; sequences were plotted in descending rank order of coverage. For example, for the E3 protein, vaccine pVXH-6 covers ~50% of WEEV isolates at a level of 82% of 9-mers or better (those to the left of the red dot), whereas 32% of isolates (those to the right of the gold dot, *x*-axis value 0.68 and above) are covered at a level of 41% or less. Colors identify candidate vaccines as in Figure 2.

Here, we evaluated coverage of published vaccine sequences against a set of reference genomes of EEEV, VEEV, and WEEV (Figure 3). Genetic variation can be summarized by the diversity of fixed-length subsequences, typically denoted as *k*-mers. Using *k* = 9 captures the potential linear epitopes recognized by adaptive immune responses, including both B and T cells [127,128,129].

We represent the coverage of potential linear epitopes as a form of cumulative distribution function, in which the proportion of vaccine-matched 9-mers varies as a function of the proportion of natural sequences (Figure 3). For the capsid, E3, and E1, the VEEV vaccine candidates had similar coverage, but for E2 and 6K, vaccines derived from ZPC738 covered VEEV isolates at a slightly higher level compared to others. No single WEEV vaccine candidate covered all five structural polyproteins better than the others. Vaccines derived from North American EEEV covered EEEV isolates at a higher level than those derived from South American Madariaga forms. Overall, no candidate vaccine could cover all of the natural sequences at a level near 100% of the 9-mers.

The coverage function decreased as more natural viral sequences were included because of increased genetic diversity among variants. Ideally, as in the case of a perfectly conserved viral protein, the fraction of 9-mers matched would remain high as more natural sequences were considered. In practice, the function decreased in a stepwise manner, reflecting the within-clade relatedness of the variants. This phenomenon is related to the known multimodal distribution of pairwise intersequence differences in the viral taxa, reflecting strains and serotypes within a viral species [130]. Cases where a vaccine candidate matches a few heterologous 9-mers in other viral species appear as lines at or near zero over the entire set of natural sequences. A rule of thumb for comparing the distributions is to consider the area under each line and taking as optimal the distribution that includes the greatest area and, hence, the greatest coverage of vaccine-matched 9-mers among known natural sequence variants.

For encephalitic Alphaviruses, coverage of the trivalent 3-EEV vaccine is depicted by a thin black line (Figure 3). It shows greater coverage for EEEV than the other vaccine candidates and slightly lower coverage for VEEV. This suggests a modified trivalent Alphavirus vaccine design may enable more broadly protective cross-reactivity.

## 8. Conclusions and Future Directions

Structural gene sequences were obtained from 32 published viral-vectored and plasmid DNA vaccines. These candidate vaccine sequences were derived from relatively few isolates: VEEV strains Trinidad donkey/TC-83 and ZPC738; WEEV strains 71V-1658, CBA87, CO92-1356, and McMillan-1941; and EEEV strains BeAr436087, FL91-4679, FL93-939, and NC_003899. This is a limited sampling from a large pool of genetic diversity. Polyvalent formulations improve the coverage of potential linear epitopes. The selection of optimal sequences in candidate EEEV, VEEV, and WEEV vaccines could improve the breath of coverage and could be useful in selecting optimal multivalent combinations for protective immunity across the equine encephalitic Alphaviruses.

Coverage of potential linear epitopes was advanced as a method to compare candidate immunogens against such highly variable pathogens as HIV-1 [127,131,132,133,134,135], HCV [136,137], and Filoviridae [138,139,140]. The approach is motivated by the high density of experimentally confirmed epitopes throughout viral proteomes, extreme diversity of known HLA alleles, and the regional variation in dominant viral strains. Rather than an over-engineer an antigen for a particular HLA type, the intent of maximizing coverage is to realize the greatest potential vaccine-induced immunity over the diversity of human populations. Driven by viral sequence data from genetic databases, it seeks to represent known diversity efficiently and is particularly useful for the design of polyvalent vaccines [141].

The currently used vaccine, TC-83, has a myriad of adverse effects and lacks full coverage of VEEV. There has been remarkable progress since the development of TC-83, including the development of investigational vaccines and the evaluation of their utility in a trivalent vaccine. Despite these advances, there are drawbacks to these vaccine candidates, which necessitate further work to move new therapeutics from the research stage in animal models to human use. Future studies should place an increased emphasis on evaluating protection against heterologous challenges. Rather than providing protection only against currently circulating strains, vaccine-design strategies that prioritize broadly protective immunity may reduce global health threats presented by newly emerging viruses and zoonotic spillovers. In addition, vaccine formulation and delivery method are important aspects to consider, as they influence safety and tolerability. Formulation and delivery methods that enable single-dose vaccines may improve compliance and are better-suited for rapid responses than multiple-dose vaccine administrations.

## Figures and Tables

**Table 1:** Summary of recent recombinant vector vaccine candidates for Eastern, Venezuelan, and Western equine encephalitis viruses.

Target	Vaccine	Genes	Animal Model	Doses, Route	Induced Immune Responses		Challenge Route	% Survival Post-Challenge		Reference
					CI ^1^	HI ^2^		Homologous	Heterologous	
**Adenovirus**										
VEEV	RAd/VEEV#3	E3-E2-6K	BALB/c mice	3, IN ^3^	NT ^4^	Yes	Aerosol	90	50–100	[67]
				2, IN	NT	Yes	Aerosol	10	NT	[68]
				3, IN	NT	Yes	Aerosol	20	NT	[69]
	RAd/VEEV#3 +CpG	E3-E2-6K	BALB/c mice	2, IN	NT	Yes	Aerosol	40	NT	[68]
	RAd/VEEV#3 -CO	E3-E2-6K	BALB/c mice	3, IN	NT	Yes	Aerosol	90	NT	[69]
WEEV	Ad5-WEEV	E3-E2-6K-E1	BALB/c mice	2, IM ^5^	NT	Yes	IN	100	NT	[70]
				1, IM	NT	Yes	IN	100	88–100	[71]
	Ad5-E1	6K-E1	BALB/c mice	1, IM	Yes	No	IN	100	0–100	[72]
**Eilat Virus**										
EEEV	EILV/EEEV	C-E3-E2-6K-E1	CD-1 mice	1, SC ^6^	NT	Yes	IP ^7^	100	NT	[73]
VEEV	EILV/VEEV	C-E3-E2-6K-E1	CD-1 mice	1, SC	NT	Yes	SC	NT	100	
EEEV, VEEV	EILV/EEEV, EILV/VEEV, EILV/CHIKV	C-E3-E2-6K-E1 and C-E2-E1	CD-1 mice	1, SC	NT	Yes	IP or SC	80	90	
**Equine Herpes Virus**										
VEEV	rH_VEEV	E3-E2-6K-E1	NIH Swiss mice	2, SC	NT	Yes	SC	NT	0–100	[74]
**Isfahan Virus**										
EEEV, VEEV	rISFV-VEEV/rISFV-EEEV	E3-E2-6K-E1	CD-1 mice	1, IM	NT	Yes	IP or SC	100	NT	[75]
**Sindbis Virus**										
EEEV	SIN/NAEEEV	C-E3-E2-6K-E1	NIH Swiss mice	1, SC	NT	Yes	IP	80–100	NT	[76]
			Cynomolgus macaques	1, SC	NT	Yes	Aerosol	82	NT	[77]
VEEV	SIN-83	C-E3-E2-6K-E1	NIH Swiss mice	1, SC	NT	Yes	SC	NT	100	[78]
				1, SC	Yes	Yes	IC ^8^ IN or SC	NT	80–100	[79]
	SAAR/TRD	C-E3-E2-6K-E1	NIH Swiss mice	1, SC	Yes	Yes	IC, IN or SC	NT	100	
			Golden hamster	1, SC	NT	NT	SC	NT	100	
	SIN/TRD	C-E3-E2-6K-E1	NIH Swiss mice	1, SC	Yes	Yes	IC, IN or SC	NT	100	
			Golden hamster	1, SC	NT	NT	SC	NT	100	
	SIN/ZPC	C-E3-E2-6K-E1	NIH Swiss mice	1, SC	Yes	Yes	IC, IN or SC	100	NT	
			Golden hamster	1, SC	NT	NT	SC	100	NT	
WEEV	SIN/CO92	C-E3-E2-6K-E1	NIH Swiss mice	1, SC	NT	Yes	IN	NT	40–100	[80]
	SIN/SIN/McM or SIN/EEE/McM	C-E3-E2-6K-E1	NIH Swiss mice	1, SC	NT	Yes	IN	100	NT	
**Vaccinia Virus**										
EEEV	MVA-BN-E	E3-E2-6K-E1	BALB/c mice	2, SC	NT	Yes	IN	NT	100	[81]
VEEV	MVA-BN-V	E3-E2-6K-E1	BALB/c mice	2, SC	NT	Yes	IN	100	NT	
WEEV	MVA-BN-W	E3-E2-6K-E1	BALB/c mice	2, SC	NT	Yes	IN	NT	100	
EEEV, VEEV, WEEV	MVA-BN-W +E+V	E3-E2-6K-E1	BALB/c mice	2, SC	NT	Yes	IN	90–100	60–100	
**Vesicular Stomatitis Virus**										
VEEV	rVSIV-VEEV	E3-E2-6K-E1	CD-1 mice	1, IM	NT	Yes	SC	100	NT	[75]

^1^ CI, cellular immunity; ^2^ HI, humoral immunity; ^3^ IN, intranasal; ^4^ NT, not tested; ^5^ IM, intramuscular; ^6^ SC, subcutaneous; ^7^ IP, intraperitoneal; and ^8^ IC, intracerebral.

**Table 2:** Summary of recent plasmid DNA vaccine candidates for Eastern, Venezuelan, and Western equine encephalitis viruses.

Target	Vaccine	Genes	Animal Model	Immunization Doses, Route	Induced Immune Responses		Challenge Route	% Survival Postchallenge		Reference
					CI ^1^	HI ^2^		Homologous	Heterologous	
EEEV	pcDNA 3.1(+)-C-E	C-E3-E2-6K-E1	BALB/c mice	3×, IM ^3^	Yes	Yes	NT ^4^	NT	NT	[104]
VEEV	26S	C-E3-E2-6K-E1	BALB/c mice	3×, gene gun	NT	Yes	Aerosol or SC ^5^	80–100	NT	[105]
	DNA-Ad	E3-E2-6K	BALB/c mice	3×, gene gun and 1×, IN ^6^	NT	Yes	Aerosol	83	NT	[106]
	AG2-5A7	E3-E2-6K-E1	BALB/c mice	3×, ID ^7^	NT	Yes	Aerosol	80	NT	[107]
	AG2-5A10	E3-E2-6K-E1	BALB/c mice	3×, ID	NT	Yes	Aerosol	70	NT	
	AG4-1C7	E3-E2-6K-E1	BALB/c mice	3×, ID	NT	Yes	Aerosol	90	NT	
	AG4-1G2	E3-E2-6K-E1	BALB/c mice	3×, ID	NT	Yes	Aerosol	100	NT	
	VEEV DNA	C-E3-E2-6K-E1	Cynomolgus macaques	3×, gene gun	NT	Yes	Aerosol	100	NT	[108]
	VEEV_CO_	E3-E2-6K-E1	BALB/c mice	2×, IM-EP ^8^	Yes	Yes	Aerosol	100	NT	[109]
				2×, IM	Yes	Yes	Aerosol	50–100	NT	[110]
			Cynomolgus macaque	2×, IM-EP	NT	Yes	Aerosol	100	NT	[109]
			Human	3×, IM-EP	NT	Yes	NT	NT	NT	[111]
	VEEV_COCAP_	C-E3-E2-6K-E1	BALB/c mice	2×, IM-EP	NT	Yes	Aerosol	90	NT	[109]
	pTC83 iDNA	Full-length cDNA	BALB/c mice	1×, IM-EP	NT	Yes	SC	NT	100	[112]
	Multi-epitope DNA	Partial sequences of C-E2-E1	BALB/c mice	3×, IM-EP	Yes	Yes	NT	NT	NT	[113]
			HLA-DR3 mice	2×, IM-EP	Yes	Yes	Aerosol	20	NT	
WEEV	pVXH-6	C-E3-E2-6K-E1	BALB/c mice	4×, gene gun	Yes	No	IN	100	50–62	[114]
				3×, gene gun	NT	NT	IN	100	50–63	[115]
	pE3-E2-6K-E1	E3-E2-6K-E1	BALB/c mice	3×, gene gun	NT	NT	IN	100	88–100	[115]
	pE3-E2	E3-E2	BALB/c mice	3×, gene gun	NT	NT	IN	0	0	
	p6K-E1	6K-E1	BALB/c mice	3×, gene gun	NT	NT	IN	100	0–75	
EEEV, VEEV, WEEV	3-EEV	E3-E2-6K-E1	BALB/c mice	2×-3×, IM-EP	Yes	Yes	Aerosol	100	NT	[116]

^1^ CI, cellular immunity; ^2^ HI, humoral immunity; ^3^ IM, intramuscular; ^4^ NT, not tested; ^5^ SC, subcutaneous; ^6^ IN, intranasal; ^7^ ID, intradermal; and ^8^ EP, electroporation.

## References

[1] Weaver S.C., Forrester N.L. (2015). Chikungunya: Evolutionary history and recent epidemic spread. Antivir. Res..

[2] Pettersson J.H.-O., Eldholm V., Seligman S.J., Lundkvist Å., Falconar A.K., Gaunt M.W., Musso D., Nougairède A., Charrel R., Gould E.A. (2016). How did Zika virus emerge in the Pacific Islands and Latin America?. MBio.

[3] Zumla A., Dar O., Kock R., Muturi M., Ntoumi F., Kaleebu P., Eusebio M., Mfinanga S., Bates M., Mwaba P. (2016). Taking forward a ‘One Health’ approach for turning the tide against the Middle East respiratory syndrome coronavirus and other zoonotic pathogens with epidemic potential. Int. J. Infect. Dis..

[4] Rauch S., Jasny E., Schmidt K.E., Petsch B. (2018). New vaccine technologies to combat outbreak situations. Front. Immunol..

[5] Chen W.H., Strych U., Hotez P.J., Bottazzi M.E. (2020). The SARS-CoV-2 vaccine pipeline: An overview. Curr. Trop. Med. Rep..

[6] Saif L.J. (2020). Vaccines for COVID-19: Perspectives, prospects, and challenges based on candidate SARS, MERS, and animal coronavirus vaccines. Eur. Med. J..

[7] Peeri N.C., Shrestha N., Rahman M.S., Zaki R., Tan Z., Bibi S., Baghbanzadeh M., Aghamohammadi N., Zhang W., Haque U. (2020). The SARS, MERS and novel coronavirus (COVID-19) epidemics, the newest and biggest global health threats: What lessons have we learned?. Int. J. Epidemiol..

[8] Gould E., Pettersson J., Higgs S., Charrel R., de Lamballerie X. (2017). Emerging arboviruses: Why today?. One Health.

[9] Bloom D.E., Black S., Rappuoli R. (2017). Emerging infectious diseases: A proactive approach. Proc. Natl. Acad. Sci. USA.

[10] Forrester N.L., Wertheim J.O., Dugan V.G., Auguste A.J., Lin D., Adams A.P., Chen R., Gorchakov R., Leal G., Estrada-Franco J.G. (2017). Evolution and spread of Venezuelan equine encephalitis complex Alphavirus in the Americas. PLoS Negl. Trop. Dis..

[11] Ronca S.E., Dineley K.T., Paessler S. (2016). Neurological sequelae resulting from encephalitic Alphavirus infection. Front. Microbiol..

[12] Villari P., Spielman A., Komar N., McDowell M., Timperi R.J. (1995). The economic burden imposed by a residual case of Eastern encephalitis. Am. J. Trop. Med. Hyg..

[13] Broeck G.T., Merrell M.H. (1933). A serological difference between eastern and western equine encephalomyelitis virus. Proc. Soc. Exp. Biol. Med..

[14] Carrera J.P., Forrester N., Wang E., Vittor A.Y., Haddow A.D., Lopez-Verges S., Abadia I., Castano E., Sosa N., Baez C. (2013). Eastern equine encephalitis in Latin America. N. Engl. J. Med..

[15] Kubes V., Rios F.A. (1939). The causative agent of infectious equine encephalomyelitis in Venezuela. Science.

[16] Suarez O.M., Bergold G.H. (1968). Investigations of an outbreak of Venezuelan equine encephalitis in towns of eastern Venezuela. Am. J. Trop. Med. Hyg..

[17] Bowen G.S., Fashinell T.R., Dean P.B., Gregg M.B. (1976). Clinical aspects of human Venezuelan equine encephalitis in Texas. Bull. Pan Am. Health Organ..

[18] Rossi A.L. (1967). Rural epidemic encephalitis in Venezuela caused by a group A arbovirus (VEE). Prog. Med. Virol..

[19] Weaver S.C., Ferro C., Barrera R., Boshell J., Navarro J.C. (2004). Venezuelan equine encephalitis. Annu. Rev. Entomol..

[20] Meyer K.F., Haring C.M., Howitt B. (1931). The etiology of epizootic encephalomyelitis of horses in the San Joaquin Valley, 1930. Science.

[21] Arechiga-Ceballos N., Aguilar-Setien A. (2015). Alphaviral equine encephalomyelitis (Eastern, Western and Venezuelan). Rev. Sci. Tech. Off. Int. Epiz..

[22] Roy C., Reed D., Hutt J. (2010). Aerobiology and inhalation exposure to biological select agents and toxins. Vet. Pathol..

[23] Wolfe D.N., Heppner D.G., Gardner S.N., Jaing C., Dupuy L.C., Schmaljohn C.S., Carlton K. (2014). Current strategic thinking for the development of a trivalent Alphavirus vaccine for human use. Am. J. Trop. Med. Hyg..

[24] Arrigo N.C., Adams A.P., Weaver S.C. (2010). Evolutionary patterns of Eastern equine encephalitis virus in North versus South America suggest ecological differences and taxonomic revision. J. Virol..

[25] Zacks M.A., Paessler S. (2010). Encephalitic alphaviruses. Vet. Microbiol..

[26] Brown R., Wan J., Kielian M. (2018). The Alphavirus exit pathway: What we know and what we wish we knew. Viruses.

[27] Olivia L.W., Obanda V., Bucht G., Mosomtai G., Otieno V., Ahlm C., Evander M. (2015). Global emergence of Alphaviruses that cause arthritis in humans. Infect. Ecol. Epidemiol..

[28] Weaver S.C., Paessler S., Barrett A.D.T., Stanberry L.R. (2009). Chapter 21—Alphaviral encephalitides. Vaccines for Biodefense and Emerging and Neglected Diseases.

[29] Cox H.R., Olitsky P.K. (1934). Prevention of experimental equine encephalomyelitis in guinea pigs by means of virus adsorbed on aluminum hydroxide. Science.

[30] Beard J., Finkelstein H., Sealy W., Wyckoff R. (1938). Immunization against equine encephalomyelitis with chick embryo vaccines. Science.

[31] Pittman P.R., Makuch R.S., Mangiafico J.A., Cannon T.L., Gibbs P.H., Peters C.J. (1996). Long-term duration of detectable neutralizing antibodies after administration of live-attenuated VEE vaccine and following booster vaccination with inactivated VEE vaccine. Vaccine.

[32] Cieslak T.J., Christopher G.W., Kortepeter M.G., Rowe J.R., Pavlin J.A., Culpepper R.C., Eitzen E.M. (2000). Immunization against potential biological warfare agents. Clin. Infect. Dis..

[33] Berge T.O., Banks I.S., Tigertt W. (1961). Attenuation of Venezuelan equine encephalomyelitis virus by ire vitro cultivation in guinea-pig heart cells. Am. J. Hyg..

[34] Kinney R.M., Johnson B.J., Welch J.B., Tsuchiya K.R., Trent D.W. (1989). The full-length nucleotide sequences of the virulent Trinidad donkey strain of Venezuelan equine encephalitis virus and its attenuated vaccine derivative, strain TC-83. Virology.

[35] Kinney R.M., Chang G., Tsuchiya K.R., Sneider J.M., Roehrig J., Woodward T., Trent D. (1993). Attenuation of Venezuelan equine encephalitis virus strain TC-83 is encoded by the 5’-noncoding region and the E2 envelope glycoprotein. J. Virol..

[36] Rayfield E., Gorelkin L., Cumow R., Jahrling P. (1976). Virus-induced pancreatid disease induced by Venezuelan equine encephalitis virus. Alterations in glucose tolerance and insulin release. J. Am. Diab. Assoc..

[37] Casamassima A.C., Hess L.W., Marty A. (1987). TC-83 Venezuelan equine encephalitis vaccine exposure during pregnancy. Teratology.

[38] Jahrling P.B., Stephenson E.H. (1984). Protective efficacies of live attenuated and formaldehyde-inactivated Venezuelan equine encephalitis virus vaccines against aerosol challenge in hamsters. J. Clin. Microbiol..

[39] Edelman R., Ascher M.S., Oster C.N., Ramsburg H.H., Cole F.E., Eddy G.A. (1979). Evaluation in humans of a new, inactivated vaccine for Venezuelan equine encephalitis virus (C-84). J. Infect. Dis..

[40] Kinney R.M., Tsuchiya K.R., Sneider J.M., Trent D.W. (1992). Molecular evidence for the origin of the widespread Venezuelan equine encephalitis epizootic of 1969 to 1972. J. Gen. Virol..

[41] Reisler R.B., Gibbs P.H., Danner D.K., Boudreau E.F. (2012). Immune interference in the setting of same-day administration of two similar inactivated Alphavirus vaccines: Eastern equine and Western equine encephalitis. Vaccine.

[42] Pittman P.R., Liu C.T., Cannon T.L., Mangiafico J.A., Gibbs P.H. (2009). Immune interference after sequential Alphavirus vaccine vaccinations. Vaccine.

[43] Steele K., Twenhafel N. (2010). Pathology of animal models of Alphavirus encephalitis. Vet. Pathol..

[44] Jackson A.C., SenGupta S.K., Smith J.F. (1991). Pathogenesis of Venezuelan equine encephalitis virus infection in mice and hamsters. Vet. Pathol..

[45] Dudley D.M., Newman C.M., Lalli J., Stewart L.M., Koenig M.R., Weiler A.M., Semler M.R., Barry G.L., Zarbock K.R., Mohns M.S. (2017). Infection via mosquito bite alters Zika virus tissue tropism and replication kinetics in rhesus macaques. Nat. Commun..

[46] Rusnak J.M., Glass P.J., Weaver S.C., Sabourin C.L., Glenn A.M., Klimstra W., Badorrek C.S., Nasar F., Ward L.A. (2019). Approach to strain selection and the propagation of viral stocks for Venezuelan equine encephalitis virus vaccine efficacy testing under the animal rule. Viruses.

[47] Höper D., Freuling C.M., Müller T., Hanke D., von Messling V., Duchow K., Beer M., Mettenleiter T.C. (2015). High definition viral vaccine strain identity and stability testing using full-genome population data—The next generation of vaccine quality control. Vaccine.

[48] Sharma A., Knollmann-Ritschel B. (2019). Current understanding of the molecular basis of Venezuelan equine encephalitis virus pathogenesis and vaccine development. Viruses.

[49] Turell M.J., Ludwig G.V., Kondig J., Smith J.F. (1999). Limited potential for mosquito transmission of genetically engineered, live-attenuated Venezuelan equine encephalitis virus vaccine candidates. Am. J. Trop. Med. Hyg..

[50] Pedersen C.E., Robinson D.M., Cole F.E. (1972). Isolation of the vaccine strain of Venezuelan equine encephalomyelitis virus from mosquitoes in Louisiana. Am. J. Epidemiol..

[51] Mueller S., Papamichail D., Coleman J.R., Skiena S., Wimmer E. (2006). Reduction of the rate of poliovirus protein synthesis through large-scale codon deoptimization causes attenuation of viral virulence by lowering specific infectivity. J. Virol..

[52] Yang C., Skiena S., Futcher B., Mueller S., Wimmer E. (2013). Deliberate reduction of hemagglutinin and neuraminidase expression of influenza virus leads to an ultraprotective live vaccine in mice. Proc. Natl. Acad. Sci. USA.

[53] Cheng B.Y., Ortiz-Riano E., Nogales A., de la Torre J.C., Martinez-Sobrido L. (2015). Development of live-attenuated arenavirus vaccines based on codon deoptimization. J. Virol..

[54] Nouen C.L., Collins P.L., Buchholz U.J. (2019). Attenuation of human respiratory viruses by synonymous genome recoding. Front. Immunol..

[55] Bull J.J. (2015). Evolutionary reversion of live viral vaccines: Can genetic engineering subdue it?. Virus Evol..

[56] Lauring A.S., Jones J.O., Andino R. (2010). Rationalizing the development of live attenuated virus vaccines. Nat. Biotechnol..

[57] Pushko P., Lukashevich I.S., Weaver S.C., Tretyakova I. (2016). DNA-launched live-attenuated vaccines for biodefense applications. Expert Rev. Vaccines.

[58] Johnson D.M., Sokoloski K.J., Jokinen J.D., Pfeffer T.L., Chu Y.K., Adcock R.S., Chung D., Tretyakova I., Pushko P., Lukashevich I.S. (2020). Advanced safety and genetic stability in mice of a novel DNA-launched Venezuelan equine encephalitis virus vaccine with rearranged structural genes. Vaccines.

[59] Tretyakova I., Tibbens A., Jokinen J.D., Johnson D.M., Lukashevich I.S., Pushko P. (2019). Novel DNA-launched Venezuelan equine encephalitis virus vaccine with rearranged genome. Vaccine.

[60] Phillpotts R.J. (2006). Venezuelan equine encephalitis virus complex-specific monoclonal antibody provides broad protection, in murine models, against airborne challenge with viruses from serogroups I, II and III. Virus Res..

[61] Parker M.D., Buckley M.J., Melanson V.R., Glass P.J., Norwood D., Hart M.K. (2010). Antibody to the E3 glycoprotein protects mice against lethal Venezuelan equine encephalitis virus infection. J. Virol..

[62] Reed D.S., Glass P.J., Bakken R.R., Barth J.F., Lind C.M., da Silva L., Hart M.K., Rayner J., Alterson K., Custer M. (2014). Combined Alphavirus replicon particle vaccine induces durable and cross-protective immune responses against equine encephalitis viruses. J. Virol..

[63] Saxena M., Van T.T.H., Baird F.J., Coloe P.J., Smooker P.M. (2013). Pre-existing immunity against vaccine vectors–friend or foe?. Microbiology.

[64] Smith G.L., Mackett M., Moss B. (1983). Infectious vaccinia virus recombinants that express hepatitis B virus surface antigen. Nature.

[65] Draper S.J., Heeney J.L. (2010). Viruses as vaccine vectors for infectious diseases and cancer. Nat. Rev. Microbiol..

[66] Price G.E., Soboleski M.R., Lo C.-Y., Misplon J.A., Quirion M.R., Houser K.V., Pearce M.B., Pappas C., Tumpey T.M., Epstein S.L. (2010). Single-dose mucosal immunization with a candidate universal influenza vaccine provides rapid protection from virulent H5N1, H3N2 and H1N1 viruses. PLoS ONE.

[67] Phillpotts R., O’brien L., Appleton R., Carr S., Bennett A. (2005). Intranasal immunisation with defective adenovirus serotype 5 expressing the Venezuelan equine encephalitis virus E2 glycoprotein protects against airborne challenge with virulent virus. Vaccine.

[68] Perkins S.D., Williams A.J., O’Brien L.M., Laws T.R., Phillpotts R.J. (2008). CpG used as an adjuvant for an adenovirus-based Venezuelan equine encephalitis virus vaccine increases the immune response to the vector, but not to the transgene product. Viral Immunol..

[69] Williams A.J., O’Brien L.M., Phillpotts R.J., Perkins S.D. (2009). Improved efficacy of a gene optimised adenovirus-based vaccine for Venezuelan equine encephalitis virus. Virol. J..

[70] Wu J.Q., Barabé N.D., Chau D., Wong C., Rayner G.R., Hu W.-G., Nagata L.P. (2007). Complete protection of mice against a lethal dose challenge of Western equine encephalitis virus after immunization with an adenovirus-vectored vaccine. Vaccine.

[71] Barabé N.D., Rayner G.A., Christopher M.E., Nagata L.P., Wu J.Q. (2007). Single-dose, fast-acting vaccine candidate against Western equine encephalitis virus completely protects mice from intranasal challenge with different strains of the virus. Vaccine.

[72] Swayze R.D., Bhogal H.S., Barabé N.D., McLaws L.J., Wu J.Q. (2011). Envelope protein E1 as vaccine target for Western equine encephalitis virus. Vaccine.

[73] Erasmus J.H., Seymour R.L., Kaelber J.T., Kim D.Y., Leal G., Sherman M.B., Frolov I., Chiu W., Weaver S.C., Nasar F. (2018). Novel insect-specific Eilat virus-based chimeric vaccine candidates provide durable, mono-and multivalent, single-dose protection against lethal Alphavirus challenge. J. Virol..

[74] Rosas C.T., Paessler S., Ni H., Osterrieder N. (2008). Protection of mice by equine herpesvirus type 1–based experimental vaccine against lethal Venezuelan equine encephalitis virus infection in the absence of neutralizing antibodies. Am. J. Trop. Med. Hyg..

[75] Nasar F., Matassov D., Seymour R.L., Latham T., Gorchakov R.V., Nowak R.M., Leal G., Hamm S., Eldridge J.H., Tesh R.B. (2017). Recombinant Isfahan virus and vesicular stomatitis virus vaccine vectors provide durable, multivalent, single dose protection against lethal Alphavirus challenge. J. Virol..

[76] Wang E., Petrakova O., Adams A.P., Aguilar P.V., Kang W., Paessler S., Volk S.M., Frolov I., Weaver S.C. (2007). Chimeric Sindbis/Eastern equine encephalitis vaccine candidates are highly attenuated and immunogenic in mice. Vaccine.

[77] Roy C.J., Adams A.P., Wang E., Leal G., Seymour R.L., Sivasubramani S.K., Mega W., Frolov I., Didier P.J., Weaver S.C. (2013). A chimeric Sindbis-based vaccine protects cynomolgus macaques against a lethal aerosol challenge of Eastern equine encephalitis virus. Vaccine.

[78] Paessler S., Fayzulin R.Z., Anishchenko M., Greene I.P., Weaver S.C., Frolov I. (2003). Recombinant Sindbis/Venezuelan equine encephalitis virus is highly attenuated and immunogenic. J. Virol..

[79] Paessler S., Ni H., Petrakova O., Fayzulin R.Z., Yun N., Anishchenko M., Weaver S.C., Frolov I. (2006). Replication and clearance of Venezuelan equine encephalitis virus from the brains of animals vaccinated with chimeric SIN/VEE viruses. J. Virol..

[80] Atasheva S., Wang E., Adams A.P., Plante K.S., Ni S., Taylor K., Miller M.E., Frolov I., Weaver S.C. (2009). Chimeric Alphavirus vaccine candidates protect mice from intranasal challenge with Western equine encephalitis virus. Vaccine.

[81] Hu W.-G., Steigerwald R., Kalla M., Volkmann A., Noll D., Nagata L.P. (2018). Protective efficacy of monovalent and trivalent recombinant MVA-based vaccines against three encephalitic alphaviruses. Vaccine.

[82] Zhang C., Zhou D. (2016). Adenoviral vector-based strategies against infectious disease and cancer. Hum. Vaccines Immunother..

[83] Lundstrom K. (2014). Alphavirus-based vaccines. Viruses.

[84] Nasar F., Palacios G., Gorchakov R.V., Guzman H., Da Rosa A.P.T., Savji N., Popov V.L., Sherman M.B., Lipkin W.I., Tesh R.B. (2012). Eilat virus, a unique Alphavirus with host range restricted to insects by RNA replication. Proc. Natl. Acad. Sci. USA.

[85] Bolling B., Weaver S., Tesh R., Vasilakis N. (2015). Insect-specific virus discovery: Significance for the arbovirus community. Viruses.

[86] Li C.-X., Shi M., Tian J.-H., Lin X.-D., Kang Y.-J., Chen L.-J., Qin X.-C., Xu J., Holmes E.C., Zhang Y.-Z. (2015). Unprecedented genomic diversity of RNA viruses in arthropods reveals the ancestry of negative-sense RNA viruses. Elife.

[87] Erasmus J.H., Auguste A.J., Kaelber J.T., Luo H., Rossi S.L., Fenton K., Leal G., Kim D.Y., Chiu W., Wang T. (2017). A Chikungunya fever vaccine utilizing an insect-specific virus platform. Nat. Med..

[88] Trapp S., von Einem J., Hofmann H., Köstler J., Wild J., Wagner R., Beer M., Osterrieder N. (2005). Potential of equine herpesvirus 1 as a vector for immunization. J. Virol..

[89] Tesh R., Saidi S., Javadian E., Loh P., Nadim A. (1977). Isfahan virus, a new vesiculovirus infecting humans, gerbils, and sandflies in Iran. Am. J. Trop. Med. Hyg..

[90] Adouchief S., Smura T., Sane J., Vapalahti O., Kurkela S. (2016). Sindbis virus as a human pathogen—Epidemiology, clinical picture and pathogenesis. Rev. Med. Virol..

[91] Overton E.T., Lawrence S.J., Wagner E., Nopora K., Rösch S., Young P., Schmidt D., Kreusel C., De Carli S., Meyer T.P. (2018). Immunogenicity and safety of three consecutive production lots of the non replicating smallpox vaccine MVA: A randomised, double blind, placebo controlled phase III trial. PLoS ONE.

[92] Tober R., Banki Z., Egerer L., Muik A., Behmüller S., Kreppel F., Greczmiel U., Oxenius A., von Laer D., Kimpel J. (2014). VSV-GP: A potent viral vaccine vector that boosts the immune response upon repeated applications. J. Virol..

[93] Geisbert T.W., Feldmann H. (2011). Recombinant vesicular stomatitis virus–based vaccines against Ebola and Marburg virus infections. J. Infect. Dis..

[94] Emanuel J., Callison J., Dowd K.A., Pierson T.C., Feldmann H., Marzi A. (2018). A VSV-based Zika virus vaccine protects mice from lethal challenge. Sci. Rep..

[95] Lee J., Kumar S.A., Jhan Y.Y., Bishop C.J. (2018). Engineering DNA vaccines against infectious diseases. Acta Biomater..

[96] Lu J., Zhang F., Xu S., Fire A.Z., Kay M.A. (2012). The extragenic spacer length between the 5′ and 3’ ends of the transgene expression cassette affects transgene silencing from plasmid-based vectors. Mol. Ther..

[97] Nelson J., Rodriguez S., Finlayson N., Williams J., Carnes A. (2013). Antibiotic-free production of a herpes simplex virus 2 DNA vaccine in a high yield cGMP process. Hum. Vaccines Immunother..

[98] Suschak J.J., Williams J.A., Schmaljohn C.S. (2017). Advancements in DNA vaccine vectors, non-mechanical delivery methods, and molecular adjuvants to increase immunogenicity. Hum. Vaccines Immunother..

[99] Garmory H.S., Brown K.A., Titball R.W. (2003). DNA vaccines: Improving expression of antigens. Genet. Vaccines Ther..

[100] Lee A.H., Suh Y.S., Sung J.H., Yang S.H., Sung Y.C. (1997). Comparison of various expression plasmids for the induction of immune response by DNA immunization. Mol. Cells.

[101] Faurez F., Dory D., Le Moigne V., Gravier R., Jestin A. (2010). Biosafety of DNA vaccines: New generation of DNA vectors and current knowledge on the fate of plasmids after injection. Vaccine.

[102] Ljungberg K., Liljestrom P. (2015). Self-replicating alphavirus RNA vaccines. Expert Rev. Vaccines.

[103] Ma J., Wang H., Zheng X., Xue X., Wang B., Wu H., Zhang K., Fan S., Wang T., Li N. (2014). CpG/Poly (I: C) mixed adjuvant priming enhances the immunogenicity of a DNA vaccine against Eastern equine encephalitis virus in mice. Int. Immunopharmacol..

[104] Boley P.A., Alhamo M.A., Lossie G., Yadav K.K., Vasquez-Lee M., Saif L.J., Kenney S.P. (2020). Porcine deltacoronavirus infection and transmission in poultry, United States. Emerg Infect. Dis..

[105] Riemenschneider J., Garrison A., Geisbert J., Jahrling P., Hevey M., Negley D., Schmaljohn A., Lee J., Hart M.K., Vanderzanden L. (2003). Comparison of individual and combination DNA vaccines for *B. anthracis*, Ebola virus, Marburg virus and Venezuelan equine encephalitis virus. Vaccine.

[106] Perkins S.D., O’Brien L.M., Phillpotts R.J. (2006). Boosting with an adenovirus-based vaccine improves protective efficacy against Venezuelan equine encephalitis virus following DNA vaccination. Vaccine.

[107] Dupuy L.C., Locher C.P., Paidhungat M., Richards M.J., Lind C.M., Bakken R., Parker M.D., Whalen R.G., Schmaljohn C.S. (2009). Directed molecular evolution improves the immunogenicity and protective efficacy of a Venezuelan equine encephalitis virus DNA vaccine. Vaccine.

[108] Dupuy L.C., Richards M.J., Reed D.S., Schmaljohn C.S. (2010). Immunogenicity and protective efficacy of a DNA vaccine against Venezuelan equine encephalitis virus aerosol challenge in nonhuman primates. Vaccine.

[109] Dupuy L.C., Richards M.J., Ellefsen B., Chau L., Luxembourg A., Hannaman D., Livingston B.D., Schmaljohn C.S. (2011). A DNA vaccine for Venezuelan equine encephalitis virus delivered by intramuscular electroporation elicits high levels of neutralizing antibodies in multiple animal models and provides protective immunity to mice and nonhuman primates. Clin. Vaccine Immunol..

[110] Suschak J.J., Bagley K., Six C., Shoemaker C.J., Kwilas S., Spik K.W., Dupuy L.C., Schmaljohn C.S. (2018). The genetic adjuvant IL-12 enhances the protective efficacy of a DNA vaccine for Venezuelan equine encephalitis virus delivered by intramuscular injection in mice. Antivir. Res..

[111] Hannaman D., Dupuy L.C., Ellefsen B., Schmaljohn C.S. (2016). A Phase 1 clinical trial of a DNA vaccine for Venezuelan equine encephalitis delivered by intramuscular or intradermal electroporation. Vaccine.

[112] Tretyakova I., Lukashevich I.S., Glass P., Wang E., Weaver S., Pushko P. (2013). Novel vaccine against Venezuelan equine encephalitis combines advantages of DNA immunization and a live attenuated vaccine. Vaccine.

[113] Bounds C.E., Terry F.E., Moise L., Hannaman D., Martin W.D., De Groot A.S., Suschak J.J., Dupuy L.C., Schmaljohn C.S. (2017). An immunoinformatics-derived DNA vaccine encoding human class II T cell epitopes of Ebola virus, Sudan virus, and Venezuelan equine encephalitis virus is immunogenic in HLA transgenic mice. Hum. Vaccines Immunother..

[114] Nagata L.P., Hu W.-G., Masri S.A., Rayner G.A., Schmaltz F.L., Das D., Wu J., Long M.C., Chan C., Proll D. (2005). Efficacy of DNA vaccination against Western equine encephalitis virus infection. Vaccine.

[115] Gauci P.J., Wu J.Q., Rayner G.A., Barabé N.D., Nagata L.P., Proll D.F. (2010). Identification of Western equine encephalitis virus structural proteins that confer protection after DNA vaccination. Clin. Vaccine Immunol..

[116] Dupuy L.C., Richards M.J., Livingston B.D., Hannaman D., Schmaljohn C.S. (2018). A multiagent Alphavirus DNA vaccine delivered by intramuscular electroporation elicits robust and durable virus-specific immune responses in mice and rabbits and completely protects mice against lethal Venezuelan, Western, and Eastern equine encephalitis virus aerosol challenges. J. Immunol. Res..

[117] Lundberg L., Carey B., Kehn-Hall K. (2017). Venezuelan equine encephalitis virus capsid-the clever caper. Viruses.

[118] Wolff J.A., Malone R.W., Williams P., Chong W., Acsadi G., Jani A., Felgner P.L. (1990). Direct gene transfer into mouse muscle in vivo. Science.

[119] Pascolo S. (2004). Messenger RNA-based vaccines. Expert Opin. Biol. Ther..

[120] Zhang C., Maruggi G., Shan H., Li J. (2019). Advances in mRNA vaccines for infectious diseases. Front. Immunol..

[121] Pardi N., Hogan M.J., Naradikian M.S., Parkhouse K., Cain D.W., Jones L., Moody M.A., Verkerke H.P., Myles A., Willis E. (2018). Nucleoside-modified mRNA vaccines induce potent T follicular helper and germinal center B cell responses. J. Exp. Med..

[122] Pardi N., Hogan M.J., Pelc R.S., Muramatsu H., Andersen H., DeMaso C.R., Dowd K.A., Sutherland L.L., Scearce R.M., Parks R. (2017). Zika virus protection by a single low-dose nucleoside-modified mRNA vaccination. Nature.

[123] Pardi N., Weissman D. (2017). Nucleoside modified mRNA vaccines for infectious diseases. Methods Mol. Biol..

[124] Geall A.J., Verma A., Otten G.R., Shaw C.A., Hekele A., Banerjee K., Cu Y., Beard C.W., Brito L.A., Krucker T. (2012). Nonviral delivery of self-amplifying RNA vaccines. Proc. Natl. Acad. Sci. USA.

[125] Samsa M.M., Dupuy L.C., Beard C.W., Six C.M., Schmaljohn C.S., Mason P.W., Geall A.J., Ulmer J.B., Yu D. (2019). Self-amplifying RNA vaccines for Venezuelan equine encephalitis virus induce robust protective immunogenicity in mice. Mol. Ther..

[126] Flower D.R., Macdonald I.K., Ramakrishnan K., Davies M.N., Doytchinova I.A. (2010). Computer aided selection of candidate vaccine antigens. Immunome Res..

[127] Fischer W., Perkins S., Theiler J., Bhattacharya T., Yusim K., Funkhouser R., Kuiken C., Haynes B., Letvin N.L., Walker B.D. (2007). Polyvalent vaccines for optimal coverage of potential T-cell epitopes in global HIV-1 variants. Nat. Med..

[128] Korber B.T., Letvin N.L., Haynes B.F. (2009). T-cell vaccine strategies for human immunodeficiency virus, the virus with a thousand faces. J. Virol..

[129] Trolle T., McMurtrey C.P., Sidney J., Bardet W., Osborn S.C., Kaever T., Sette A., Hildebrand W.H., Nielsen M., Peters B. (2016). The length distribution of class I-restricted T cell epitopes Is determined by both peptide supply and MHC allele-specific binding preference. J. Immunol..

[130] Van Regenmortel M.H. (2007). Virus species and virus identification: Past and current controversies. Infect. Genet. Evol..

[131] Kong W.P., Wu L., Wallstrom T.C., Fischer W., Yang Z.Y., Ko S.Y., Letvin N.L., Haynes B.F., Hahn B.H., Korber B. (2009). Expanded breadth of the T-cell response to mosaic human immunodeficiency virus type 1 envelope DNA vaccination. J. Virol..

[132] Barouch D.H., O’Brien K.L., Simmons N.L., King S.L., Abbink P., Maxfield L.F., Sun Y.H., La Porte A., Riggs A.M., Lynch D.M. (2010). Mosaic HIV-1 vaccines expand the breadth and depth of cellular immune responses in rhesus monkeys. Nat. Med..

[133] Santra S., Liao H.X., Zhang R., Muldoon M., Watson S., Fischer W., Theiler J., Szinger J., Balachandran H., Buzby A. (2010). Mosaic vaccines elicit CD8+ T lymphocyte responses that confer enhanced immune coverage of diverse HIV strains in monkeys. Nat. Med..

[134] Korber B., Fischer W. (2020). T cell-based strategies for HIV-1 vaccines. Hum. Vaccines Immunother..

[135] Stephenson K.E., Wagh K., Korber B., Barouch D.H. (2020). Vaccines and broadly neutralizing antibodies for HIV-1 prevention. Annu. Rev. Immunol..

[136] Yusim K., Fischer W., Yoon H., Thurmond J., Fenimore P.W., Lauer G., Korber B., Kuiken C. (2010). Genotype 1 and global hepatitis C T-cell vaccines designed to optimize coverage of genetic diversity. J. Gen. Virol..

[137] Yusim K., Dilan R., Borducchi E., Stanley K., Giorgi E., Fischer W., Theiler J., Marcotrigiano J., Korber B., Barouch D.H. (2013). Hepatitis C genotype 1 mosaic vaccines are immunogenic in mice and induce stronger T-cell responses than natural strains. Clin. Vaccine Immunol..

[138] Fenimore P.W., Muhammad M.A., Fischer W.M., Foley B.T., Bakken R.R., Thurmond J.R., Yusim K., Yoon H., Parker M., Hart M.K. (2012). Designing and testing broadly-protective filoviral vaccines optimized for cytotoxic T-lymphocyte epitope coverage. PLoS ONE.

[139] Theiler J., Yoon H., Yusim K., Picker L.J., Fruh K., Korber B. (2016). Epigraph: A vaccine design tool applied to an HIV therapeutic vaccine and a pan-Filovirus vaccine. Sci. Rep..

[140] Rahim M.N., Wee E.G., He S., Audet J., Tierney K., Moyo N., Hannoun Z., Crook A., Baines A., Korber B. (2019). Complete protection of the BALB/c and C57BL/6J mice against Ebola and Marburg virus lethal challenges by pan-Filovirus T-cell epigraph vaccine. PLoS Pathog..

[141] Abdul-Jawad S., Ondondo B., van Hateren A., Gardner A., Elliott T., Korber B., Hanke T. (2016). Increased valency of conserved-mosaic vaccines enhances the breadth and depth of epitope recognition. Mol. Ther..

